# Analysis and Visualization of 3D Motion Data for UPDRS Rating of Patients with Parkinson’s Disease

**DOI:** 10.3390/s16060930

**Published:** 2016-06-21

**Authors:** Neltje E. Piro, Lennart K. Piro, Jan Kassubek, Ronald A. Blechschmidt-Trapp

**Affiliations:** 1 Institute of Medical Engineering and Mechatronics, Ulm University of Applied Sciences, Albert-Einstein-Allee 55, Ulm D-89081, Germany; blechschmidt-trapp@hs-ulm.de; 2 Faculty of Physics, Ludwig-Maximilians-Universität München, Geschwister-Scholl-Platz 1, Munich D-80539, Germany; lennart.piro@campus.lmu.de; 3 Department of Neurology, University of Ulm, Oberer Eselsberg 45, Ulm D-89081, Germany; jan.kassubek@uni-ulm.de

**Keywords:** MARG sensors, inertia sensors, IMU, motion data, Parkinson’s Disease, UPDRS, symptom quantification, animated 3D avatar, telemonitoring, remote monitoring, pronation-supination, diadochokinesis

## Abstract

Remote monitoring of Parkinson’s Disease (PD) patients with inertia sensors is a relevant method for a better assessment of symptoms. We present a new approach for symptom quantification based on motion data: the automatic Unified Parkinson Disease Rating Scale (UPDRS) classification in combination with an animated 3D avatar giving the neurologist the impression of having the patient live in front of him. In this study we compared the UPDRS ratings of the pronation-supination task derived from: (a) an examination based on video recordings as a clinical reference; (b) an automatically classified UPDRS; and (c) a UPDRS rating from the assessment of the animated 3D avatar. Data were recorded using Magnetic, Angular Rate, Gravity (MARG) sensors with 15 subjects performing a pronation-supination movement of the hand. After preprocessing, the data were classified with a J48 classifier and animated as a 3D avatar. Video recording of the movements, as well as the 3D avatar, were examined by movement disorder specialists and rated by UPDRS. The mean agreement between the ratings based on video and (b) the automatically classified UPDRS is 0.48 and with (c) the 3D avatar it is 0.47. The 3D avatar is similarly suitable for assessing the UPDRS as video recordings for the examined task and will be further developed by the research team.

## 1. Introduction

Parkinson’s disease (PD) is an age-related, neurodegenerative disorder whose underlying pathological processes can be traced as a topographically ascending degeneration scheme spreading from the lower brainstem toward mesencephalic structures and the basal ganglia, finally reaching the neocortex, as evident from neuropathological studies [1]. The classical cardinal symptoms—namely bradykinesia, rigidity, tremor, and postural instability—affect the motor skills of the patients [2]. In daily clinical practice, the severity of PD-related symptoms are typically quantified using the Unified Parkinson Disease Rating Scale (UPDRS) revised by the Movement Disorder Society (MDS) in 2003 [3]. Part III of the UPDRS considers the examination of motor symptoms. Various items are rated on a five-point rating scheme, from 0 for no impairment to 4 for highest severity. The full assessment of the patient’s health status with the UPDRS is time-consuming, is dependent on the rater’s experience [4], and considers only a snapshot of time. The assessment of motor function over time is necessary for an individual adjustment of medication and appropriate quality of treatment. To assess motor fluctuations during the day, patient diaries or questionnaires are typically used. These tools give the doctor a basic idea of the domestic situation but they remain subjective and strongly dependent on the patient’s capabilities and compliance.

Neurologists and PD patients would thus benefit from the continuous monitoring of symptoms in everyday life. There have been many attempts to reach an objective, rater-independent assessment of symptoms in the daily life of PD patients. For the quantification of motor symptoms video cameras and motion sensors are used. Video-based systems allow the physician a visual impression of the patient but have the drawback that the assessment is restricted to one location and motor tasks have to be analyzed manually. This makes the evaluation of long-term trends of the patient’s condition disproportionally time-consuming. Video recordings also give insight into the living conditions of the patient, which can result in limited patient acceptance. In contrast, motion sensors can be worn on the body and capture symptoms in everyday situations. Motion sensors include accelerometers and/or gyroscopes and/or magnetic field sensors for drift compensation. If all of these sensors are contained in one unit, it is also called a Magnetic, Angular Rate, Gravity (MARG) sensor. The evaluation of symptoms with motion sensors can be based either on defined motor tasks [5,6,7,8,9,10,11,12] that the patients need to perform periodically during their daily routines, or continuously in everyday movements [13,14,15]. The collected raw data must be processed in a way that allows for their use in clinical decision support. Most systems use machine learning techniques to objectively predict the UPDRS score [7,8,9,12,13,16,17] or their own continuous metrics [11] from data features. The results are usually presented in the form of tables or charts.

In order to achieve a high user acceptance, it must be ensured that the data can be easily interpreted by neurologists and to provide exactly the information that is needed to derive clinical decisions. Therefore, it is important that the neurologists are familiar with the given information from their clinical routine. For motor assessment, neurologists apply the UPDRS, as a quantitative scoring system, based on the visual impression of their patients. Previous work from other authors can provide either a visual impression by video recordings or compute the UPDRS score by MARG sensor data, but not both. To convey both impressions, the two measuring systems would have to be combined. Hence we present a new approach giving both impressions using only MARG sensors for home monitoring.

### Project Vision and Goals

This work is part of a research project [18] at Ulm University of Applied Science in Germany. The aim of the project is the development of a telemonitoring system for PD patients using MARG sensors. The system consists of multiple sensor units, an Android application for interaction and feedback to the patient, as well as an application server providing a web browser application to the neurologist for the analysis of patient data. In addition to a quantitative data analysis by UPDRS, gathered motion data should be visualized as an animated 3D human avatar. This avatar should move as naturally and realistically as possible to give the physician (neurologist) the impression of having the patient live in front of him. In contrast to video recordings of patients in the domestic environment, the data recording for the avatar animation is not bound to a specific location and does not allow any insight into the private life of the patient.

The overall concept of the telemonitoring system envisages using the UPDRS classifications for an overview of the long-term condition. This should provide neurologists with a quick impression of the course of symptoms over the monitoring period and fluctuations during the day (”all information at a glance“). In addition, the avatar allows the neurologist to assess the patient’s movements in specific situations, e.g., situations in which the patient complained about unusually severe symptoms. Hence the avatar supplements the calculated UPDRS by the visual impression. Having both, the neurologist can derive a substantiated clinical decision. This concept is, to our knowledge, a novel approach which has not been described in the literature.

This system concept will be evaluated exemplarily regarding one motor task in a clinical setting. The goal of this study is to compare the UPDRS ratings resulting from: (a) an examination based on video recordings as clinical reference; (b) an automatically computed UPDRS with a classification algorithm as method in home monitoring with MARG sensors; and (c) a UPDRS rating on the basis of a 3D animated human avatar. The results should show, whether the 3D avatar is suited for UPDRS rating and how the ratings are associated with each other.

## 2. Material and Methods

**Figure 1:**
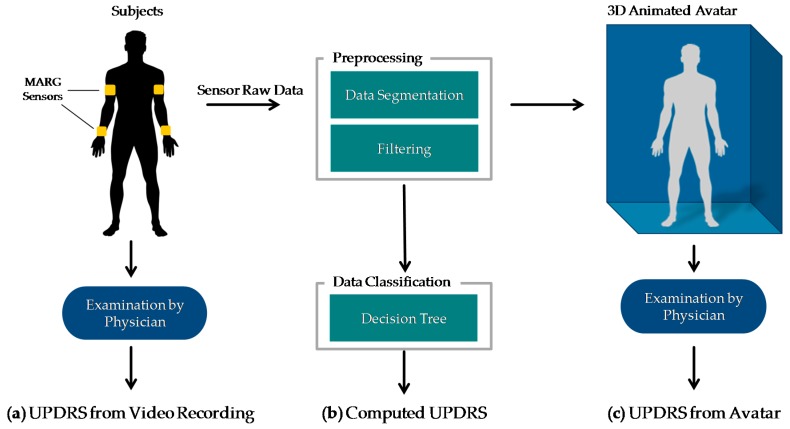
Comparison of UPDRS ratings based on (**a**) Video recordings; (**b**) Automated classification algorithm; (**c**) Animated 3D avatar.

The methods of this work are summarized in Figure 1. PD patients as well as controls performed item 3.6 “Pronation-supination movements of hands” of the MDS-UPDRS [19] wearing four MARG sensors on their wrists and upper arms (figured as yellow blocks in Figure 1a). The movements of the patients were: (a) examined in video recordings by neurologists with a specific expertise in movement disorders and rated by UPDRS. These ratings were taken as clinical reference. The raw motion data were preprocessed which involved filtering, segmentation, extraction of the sensor orientation, and feature generation. The preprocessed data were then used for (b) an automated computation of the UPDRS using data classification techniques and (c) the generation of an animated 3D avatar. The avatar was examined by the same movement disorders specialists and rated by UPDRS as well. The resulting UPDRS ratings of methods a, b and c were compared on a common evaluation data set. The following sections give a closer look at the details of these steps and the evaluation methodology. All data gathered in this study and the computed features with their mathematical description as well as the results of the different UPDRS ratings can be found on Zenodo ([20], see Appendix A).

### 2.1. Sensor System

**Figure 2:**
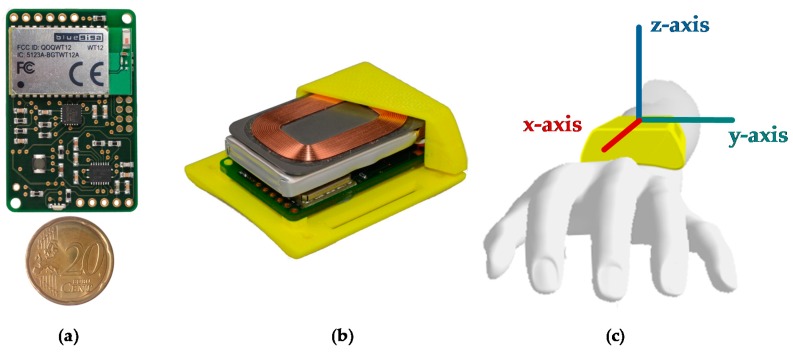
The sensor board (**a**) in comparison with a 20 cent coin and (**b**) in the casing; (**c**) axis of the sensor unit placed on the avatar’s arm.

A sensor unit was entirely self-developed at Ulm University of Applied Sciences for the monitoring of PD patients. The sensor unit comprises of a 9-axis MARG sensor (MPU-9150 from InvenSense, San Jose, CA, USA) with a ±4 g @16 Bit, ±2000°/s @16Bit, and ±1200 µT @13Bit measuring range and a sampling rate of up to 200 Hz. The small, ergonomically shaped case is 48 mm × 42 mm × 15 mm and manufactured with rapid prototyping. The total weight of the sensor unit is 32 grams. Figure 2a shows the size and Figure 2b the inner components: printed circuit board, battery, and charging coil. The sensor unit can be attached to the arm or leg by an elastic band. Recorded data can be sent via Bluetooth to a gateway device (e.g., smartphone) or stored on an integrated flash memory if the connection is lost. The unit is powered by a 600 mAh Li-Pol battery, yielding a typical run time of 14 h.

### 2.2. Subjects and Data Collection

Data were collected between June and July 2015 in the outpatient clinic of the Department for Neurology, University of Ulm. All participants were notified of the study’s procedure and goals before signing an informed consent form. The inclusion criterion for subjects was a clinical diagnosis of Parkinson’s disease (UK Brain Bank Clinical Diagnosis Criteria [2]) and motor symptoms for a duration of at least two years, while controls should not have been diagnosed with parkinsonism or any other neurological diseases. All participants filled out a short questionnaire with person-related master data. The PD patients answered the PDQ-8 questionnaire [21]. This questionnaire is a short form of the 39-item Parkinson’s Disease Questionnaire (PDQ-39) [22] for the quality of life of PD patients. Based on the answers of eight questions a scale from 0–100 is computed, where 0 stands for no problems and 100 for maximum level of problems. The short form was chosen because it was less time-consuming for the subjects and has comparable explanatory power regarding overall health status [21].

Table 1 summarizes the descriptive statistical parameters of the study population. The age and sex of the two groups were mean-tested (Wilcoxon-Mann-Whitney test, two independent samples *t*-test) and were found not to be statistically different at the 5% significance level. Therefore, the groups can be considered age- and sex-matched.

All study volunteers were asked to perform the pronation-supination task from “Part III: Motor Examination” of the MDS-UPDRS with each hand separately. Sensor units were placed on the wrist and the upper arm of both body sides (compare Figure 1a). The exercise started from a hand resting position on the knees. Then each subject had to lift one arm into a horizontal position, “turn the palm up and down alternating 10 times as fast and as fully as possible” [19] and lower the arm to the resting position on the knee again. The task—as indicated in MDS-UPDRS—was demonstrated to the study subjects before they performed the task twice per body site. Movement data from the four sensors were sampled at 100 Hz and transmitted to a desktop recording software via Bluetooth. The execution of the tasks was recorded with a video camera in front of the participants. The complete protocol took 20 min on average per subject. In Table 2, the criteria for task scoring can be found. The control group had a mean UPDRS score of 0.51 (see Table 1) meaning that even some controls performed the task in a way that raters classified as “mildly severe”.

### 2.3. Data Preprocessing

According to the guidelines of the MDS, three movement characteristics should be evaluated for scoring Task 3.6: rhythm, speed, and amplitude decrement (see Table 2). These characteristics had to be extracted from the recorded data sets.

First, raw data was low-pass filtered and the orientation was computed by applying the Madgwick filter [23], developed by Madgwick in 2010. It is a Kalman-based filter, which estimates the orientation by fusing the long-term drift-free gravitation and earth magnetic field with the precise, but drifting, angular rate from the gyroscopes. A filter gain β equal to 0.041 was used as proposed in [23]. Advantages of the Madgwick filter are high performance drift compensation with low computational load and simple tuning of the filter gains.

**Figure 3:**
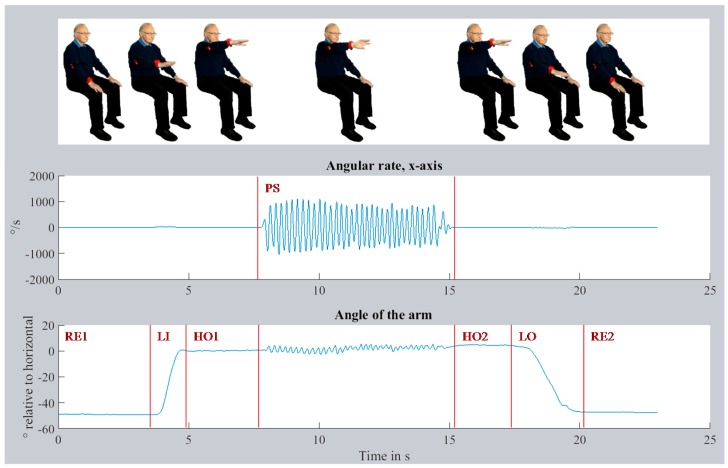
Visualization of the pronation-supination task and segmentation of the movement data.

The segmentation of the recorded data was automated with a SVM (Support Vector Machine) using the MATLAB toolbox Classification Learner. The SVM was evaluated and successfully validated in a previous investigation. Seven phases were defined for the segmentation: initial resting position on the thigh (RE1), lifting the arm (LI), keeping the arm steady in horizontal position (HO1), pronation-supination for at least 10 turns (PS), keeping the arm steady in a horizontal position (HO2), lowering of the arm (LO), and finally resting position on the thigh (RE2). The movement task with data and still frames of all phases are visualized in Figure 3. The figure shows the angular rate along the x-axis of the lower arm and the lift by approx. 45° (Euler angle relative to horizontal) of the arm. From the PS phase, 51 time- and frequency-based features were computed. Seven features were based on the acceleration data of the lower arm sensor data, 16 features on the orientation of upper and lower arm, and the remaining 28 on the angular rate data of the x-axis (along the axis of the lower arm, see Figure 2c for the coordinate system). A detailed mathematical description of all features is given on Zenodo [20].

Eight of 51 features were selected by a feature selection algorithm for the classification task (see Section 2.4). The correlation between these features and the video-based UPDRS score of the 86 training data sets was computed using the Pearson correlation coefficient. The coefficients and details about the eight features are given in Table 3. All selected features were computed after the separation of single oscillations (pronation-supination movement). For this purpose, all zero crossings of the PS phase were determined. Data were split into oscillation pair-segments each consisting of two adjacent oscillations, so that all oscillations belong to two segments, except for the first and last one. All computations were first done for each pair-segment separately, then features were derived from these computations using various descriptive statistical functions (mean, SD, quantiles, *etc.*).

Basically, three different types of computations were done. Firstly, the angular rate and its integral were used to describe speed of movement (hand turnings) and the angle of the rotation reached (*mean_angRate*, *median_rotAngle*, *upQuart_rotAngle*). Secondly, the speed and angle of rotation at the beginning of the task in relation to the end of the task were determined in order to observe fatigue (*ratioQ13_AngRate*, *ratioQ13_rotAngle*). For these, the number of hand rotations were divided into thirds, and the ratio between the mean of the first third and the last third were computed. Hence, a value below one indicated a decrement of the feature during the test. Thirdly, using a curve fitting, three features (*std_rsquare_1n*, *mean_rsquare_3n*, *std_rsquare_3n*) were computed, which reflect the rhythm or rather the precision of the movement. They were based on the sum of sines model, see Equation (1):

(1) $$y=\overset{n}{\underset{i=1}{\sum }}a_{i}\sin\left(b_{i}x+c_{i}\right)$$

Each oscillation pair-segment was fitted to a single-term (*n* = 1) and three-term (*n* = 3) sine function, corresponding to the features ending in “_1n” and “_3n”, respectively. The coefficient of determination (*R*²) of each fit in each pair-segment was computed. The standard deviation reflects the variation of the single oscillations from a rhythmic sinusoidal movement or, in other words, the rhythm. The precision of the oscillations was indicated by the two features based only on the three-term sine fit (mean_rsquare_3n, std_rsquare_3n). For all oscillation pair-segments, the mean and standard deviation of *R*² values of the third order fit are computed.

### 2.4. Computation of UPDRS

An important component of the system is the association of a measured set of features with a specific UPDRS score. Therefore, a classification algorithm was trained and validated by employing the Waikato Environment for Knowledge Analysis (WEKA, Version 3.6.16) from the University of Waikato. Each subject performed the pronation-supination motor task three or four times, resulting in 101 data sets. 15 data sets were randomly chosen as common evaluation data for the comparison of the three rating methods (compare Figure 1). The remaining 86 data sets were used for training. Information about training and evaluation data are shown in Table 4.

An experienced rater (Rater D) was asked to score the training data on the basis of the video recordings. These ratings were used as class labels for the classification task. In WEKA, the features were filtered using a supervised feature selection algorithm (BestFirst, greedy hillclimbing with backtracking [24]) and were thus reduced from 51 to nine features. For classification, the J48 tree algorithm was chosen because it showed good classification results on the data. J48 is an open source Java implementation of the C4.5 algorithm developed by Quinlan [25]. It builds a decision tree following a recursive divide-and-conquer approach to split nodes into a subset of two partitions and on the basis of information entropy it decides how nodes should be arranged in the decision tree. The J48 algorithm was trained on training data. The resulting decision tree was transformed to source code automatically through WEKA and stored as a Java source code file for later use. Finally, the predictive model was tested against the independent evaluation data set in comparison to the ratings based on the videos and the 3D avatar.

### 2.5. Generation of 3D Avatar

The requirements on a framework for implementing the 3D animated avatar were threefold. One was the processing of quaternions. Another was the possibility of displaying the animation in a web browser for evaluation by a neurologist, and on an Android smartphone for feedback to the patients. A third requirement was that the framework was available under an open source license. The Java based libGDX framework (v1.5.4) [26] was selected for the development of the 3D-Avatar in the Eclipse IDE (Luna).

The 3D human model was created with the open source tool Make Human (v1.0.2) [27]. All modelling settings (e.g., gender, age, muscle, height) were averaged to create a neutral human. The model was rigged using the preset humanik.json, resulting in a skeleton with 65 bones. Before loading the model to libGDX it was brought to sitting position in Blender (v2.73) and seated on a cube representing a chair. The 3D environment (floor and walls) was created in Blender. Finally, the model was exported as g3d-file for use in libGDX.

For the animation in libGDX, the camera position and light position were switched depending on the side of the arm movement, in order to gain maximum recognizability and contrast. The side of movement can automatically be computed from the integral of the angular rate on the x-axis (rotation angle), because supination from the horizontal position has a typical angle of +120° which is higher than the pronation with an angle of +30°. Then, orientation data of both the upper and lower arm were applied to the corresponding two bones. The movement of the upper arm moves the origin of the lower arm. Hence, the upper arm movement had to be subtracted from the lower arm movement. A rendering frequency of 60 frames per second (twice the standard video sampling rate) was chosen for smooth movement. Thus, orientation data at a sampling rate of 100 Hz was spline-interpolated and resampled to 60 Hz.

### 2.6. Evaluation of UPDRS Ratings

Fifteen data sets, from 10 PD patients and five controls, were randomly chosen for the evaluation of the avatar. The raw data of the evaluation data set (see Table 4) were preprocessed and 3D avatar animations were generated. The faces of all participants in the video recordings were pixelated to ensure that patients were not recognized by their neurologists. The avatar animations and video sequences were put in random order. Six experienced movement disorder neurologists from the clinic for neurology, who rate the UPDRS at least several times a week, were asked to rate the avatar animations and video sequences. To ensure that all neurologists used the same criteria for rating (see Table 2), the criteria were printed and provided to all raters. After rating, the neurologists filled out a short questionnaire about their impression of the avatar animations in contrast to a personal or video-based examination of the patients. To summarize, each of the evaluation data sets was rated: (a) on the basis of the video recording; (b) by the classification algorithm and (c) based on the avatar animation (compare Figure 1). The statistical analysis of this data was performed with SAS (SAS Institute Inc., Cary, NC, USA).

## 3. Results

**Figure 4:**
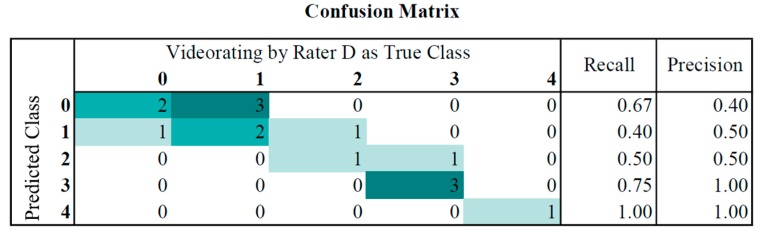
Confusion matrix of the implemented classification algorithm compared to predefined classes.

For classification, the J48 algorithm was trained on 86 data sets using WEKA. It built a pruned tree with 8 of 9 features. Evaluating the predictive model against the common evaluation dataset produced the following results: the weighted true positive (TP) rate of 0.6 and weighted false positive (FP) rate of 0.198, with a precision of 0.647. In Figure 4 the confusion matrix for the classification results, in comparison to the video ratings of Rater D, is shown. The classifier had a maximum difference of 1 and tended to underestimate the UPDRS score (mean 1.4) in comparison to Rater D (mean 1.7).

**Figure 5:**
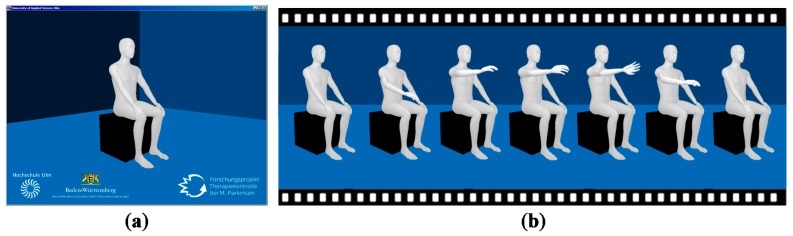
Implemented 3D avatar (**a**) Full screen view; (**b**) Pronation-supination movement sequence.

The implemented 3D avatar is presented in Figure 5. The avatar was designed so that it allows no indication of age, gender or ethnic group of the patient. Clothing was also dispensed with in order to draw the focus of the rater to the movements of the avatar. The chair and the background were also kept simple for those reasons. The deployed version of libGDX did not support the generation of shadows. Hence, only light spots were implemented for good contrast. The avatar has been optimized for desktops and has not been tested on Android or in a web browser so far. On the desktop, the avatar makes smooth movements and does not jiggle.

**Figure 6:**
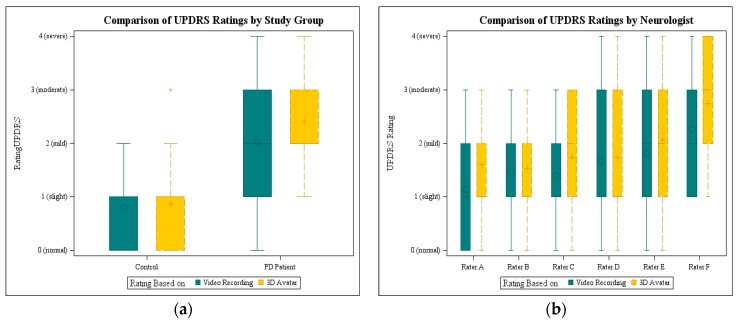
Boxplots visualizing the comparison of UPDRS ratings (**a**) by study group and (**b**) by rater.

As a first evaluation of the avatar, it was presented to the six neurologists and compared to video recordings. Each rater scored 15 video recordings and 15 animations. The ratings of all neurologists were statistically analyzed using the Kruskal-Wallis-ANOVA. There was no statistically significant difference between video and avatar ratings (*p* = 0.14), when all raters were taken into account. In Figure 6a the results are shown grouped by rating base per study group. The mean for controls is nearly the same (0.8 video and 0.87 avatar). The impairment of PD patients was rated higher on the basis of the 3D avatar (2.03 video and 2.42 avatar), but with a smaller range. In contrast to the video recordings, none of the PD avatars was rated with a normal UPDRS score 0.

Considering the ratings of each neurologist, it was noticeable that only two neurologists used the whole range from 0 to 4. The plots with the video ratings and avatar ratings per neurologists are displayed in Figure 6b. There was a significant difference between the raters (*p* = 0.0039, Kruskal-Wallis ANOVA) regarding avatar and video ratings. The individual neurologists rated consistently so that there were no significant differences between the ratings based on video and avatar, considering each rater.

**Figure 7:**
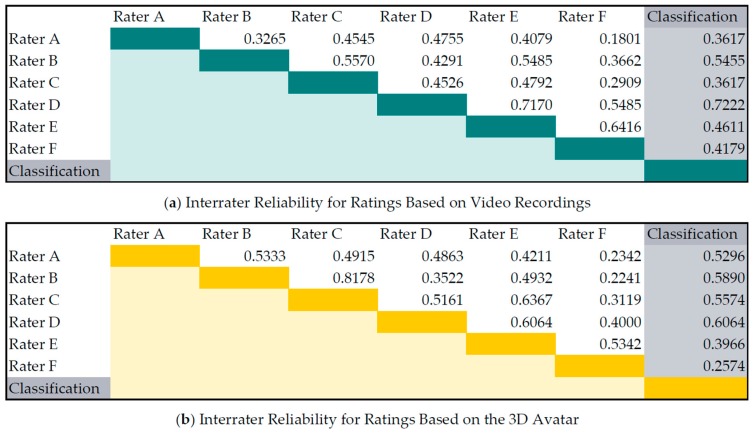
Interrater reliability for ratings based on (**a**) video recordings and (**b**) animated 3D avatar. Reliability is computed with weighted kappa coefficient.

In Figure 7 the interrater reliability between each rater as well as between the raters and the classification algorithm are shown. The reliability was computed with weighted Cohen’s kappa statistics. The agreement between the video ratings were comparable with the ratings based on the 3D avatar. Rater A and Rater F had the lowest mean agreement with the other raters. The ratings produced by the classification algorithm are comparable to those given by the raters, with a mean agreement of 0.48 with the ratings based on videos resp. 0.47 with the ratings based on the 3D avatar. The classifier had the highest agreement (videos 0.72 resp. avatar 0.61) with Rater D, who rated the training data.

**Figure 8:**
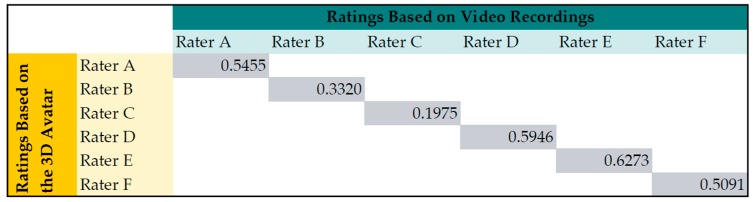
Intrarater reliability for ratings based on video recording and animated 3D avatar. Reliability is computed with weighted kappa coefficient.

Looking at the kappa statistics for avatar and video recording per rater (see Figure 8), the mean agreement is 0.48. This was comparable to the agreement between the raters. Rater C had the smallest correlation between his ratings based on video and avatar. Nevertheless, there was no significant difference (Wilcoxon-Mann-Whitney test) for the scores based on avatar and video of Rater C.

## 4. Discussion

The aim of this study was the comparison of UPDRS ratings obtained in three different ways: (a) on the basis of video recordings; (b) automatically computed using data mining techniques and (c) rated on the basis of an animated 3D avatar. It has been shown that the difference between ratings based on the 3D avatar and from ratings based on video recording is statistically non-significant. The reliability of the classifier is comparable to the ratings between different neurologists.

The system concept of combining an automatic UPDRS classification with an animated 3D avatar was evaluated in a clinical setting. Video recordings were used as clinical reference. Although the assessment of videos is not the gold standard in clinical routine, they were necessary to make clinical evaluation of the motor task possible for all six raters. This methodology has also been applied by other research teams (e.g., [5,11,16,17]). Video recordings will not be used in the prospective telemonitoring system, because the recording is bound to a specific location and videos provide insight into the patients’ private lives. Primarily, the neurologists will be provided with the computed UPDRS scorings, which they know from clinical routine. It is computed from various features that reflect different movement characteristics. In general, the features allow a more differentiated evaluation of the patient’s condition and are independent from rating patterns. However the interpretation needs a certain knowledge of the sensor and the mathematical evaluation. Moreover, some features must be interpreted in correlation with each other (e.g., a fast movement is only assessed positively when the patient has a high amplitude with a regular movement). This makes the UPDRS score a more suitable metric for the remote assessment of motor tasks. It has to be investigated if the analysis of long-term trends may benefit from the use of single features instead of the five-stage scale. The features could be a valuable enhancement to assess the treatment efficacy and help make fundamental decision, such as whether a neurostimulator is to be implanted or not.

When evaluating the videos it became apparent that each of the six neurologists has a slightly different way of rating the specific UPDRS item. One neurologist reported that he is instructing the patients to turn their hand as fast as they can while another neurologist wants smooth and proper rotations. Another neurologist mentioned that he instructs the patients to do more than 10 turns to observe signs of fatigue. These different rating patterns are noticeable in the scoring range of the neurologists and the interrater reliabilities. The agreement between Raters A and F is very poor (Weighted kappa 0.18) which can be interpreted as almost random agreement. Yet, considering the ratings based on video and avatar, each neurologist seemed to evaluate consistently in his own pattern. Prospectively, the avatar could be used to adapt the UPDRS classification to the rating pattern of a single neurologist using self-learning algorithms. This would require the neurologist to rate a sufficient number of 3D animations, leading to a slow adaption of the automatically generated score to the individual rater’s pattern of the neurologist. An adaption of the rating pattern could increase the confidence of neurologists using the system but would mean that the individually trained scores would no longer be comparable. Hence, the system could provide an individual UPDRS and a standard UPDRS for comparison. It is conceivable that in the future, not only the neurologists but also patients could benefit from the avatar on their smartphones. The avatar could reflect the patient’s movements and, combined with the UPDRS rating, allow prompt feedback about their health situation on their smartphone.

Regarding the automated UPDRS scoring, the classifier showed a good result with accuracy of 88%. The J48 classifier has been found to be suitable for this task and capable of distinguishing between the five classes (UPDRS 0–4). The test, on agreement between classification results and neurologists by interrater reliability, showed that the algorithm was comparable to the agreement between different raters. Nevertheless, the classes in the training data set were not uniformly distributed, which would have been ideal for training. This is why the classifier tends to concentrate the predicted UPDRS values in correspondence with the dominant UPDRS classes. Furthermore, it is not ideal that data sets from the same subjects were used for training and evaluation. If the classification model is overfitted to these patients, this could lead to overly good classification results. The results should be reviewed with a larger evaluation set of different subjects to draw reliable conclusions.

The classifier was trained using eight from initially 51 computed features. In contrast to Patel *et al.* [5], who used general features not linked to a specific exercise, we focused on the pronation-supination motor tasks and developed features adapted particularly to this task. While other groups also assessing the PS task applied rectangular windows with constant length [5,10], we computed features based on two full oscillations of varying duration, the oscillation pair-segments. The mean values of the features for both alternatives were nearly identical, e.g., the Pearson correlation coefficient between the RMS value of the full phase compared to the average of the segmented oscillations (*mean_angRate*) was 0.99 for the training dataset with *n* = 86. However, the oscillation-based analysis allows one to assess the form of individual arm movements, and additionally the change of the movements over time. For example, the standard deviation of the sine fits (*std_rsquare_1n* and *std_rsquare_3n*) characterizes the rhythm and smoothness as well as regularity of the pronation-supination. So far, the classifier has only been trained with features from the pronation-supination phase. In future studies, features from other phases, e.g., the lifting and lowering slope, could also turn out to be useful. As lifting and lowering are not part of the pronation-supination task, the features could be used for scoring other items. Examples are the spontaneity of movement (see MDS-UPDRS 3.14) from the lifting and lowering phase, the resting tremor in the initial resting position, as well as the postural tremor in horizontal positions (see MDS-UPDRS 3.15 to 3.18). Several UPDRS items could be estimated from one movement task lasting 20–30 s that the patient performs during the day, at home. That would be very comfortable and effective for the patient.

In the course of this project, we developed a 3D avatar in libGDX. The animated 3D avatar should be used to give a visual impression about the patient: with the avatar, it is possible to present only relevant data for scoring a single task. The avatar allows the neurologist to get a detailed impression of the movements and which characteristics were decisive for the UPDRS classification. Currently, the avatar has to be generated manually. A user has to preprocess data with MATLAB and then load the data files in to our Java desktop software. In a productive system, this process would be automated on the server receiving data from the patient, processing it and generating the avatar requested by the neurologist. The avatar was presented to six neurologists and rated by UPDRS. The scores based on the animated avatar were comparable to those based on video recordings although the neurologists were not trained in rating the 3D avatar. The neurologists were asked about their impressions of the 3D avatar. They expected that they would rate single items of the UPDRS in a more objective manner, because impressions irrelevant to the task of the patient, like posture or sitting in a wheelchair, would not alter their judgment. This estimation will be reviewed in a future study. In the questionnaire for the neurologists, they evaluated the 3D avatar as well-suited to UPDRS ratings in comparison to a personal or video-based examination of the patients. Some remarked on the relatively low contrast between the hand and body of the avatar. One reason for the weak contrast was the lack of shadows. This could, as a first step, be improved by using the new version 1.7.1 of libGDX, where shadow-rendering is added. Further possibilities would be to use different colors for the palm and back of the hand as well as for the body. One neurologist suggested showing only the avatar’s arm without the body. This would lead to a less realistic impression of the whole patient but would set the focus to the relevant body part. In addition, the omission of the avatar’s body would solve another technical issue: Depending on thorax height and arm length of the patient, the angle of the limbs between resting position and horizontal can be different from the one used for the avatar. This leads to the effect that either the avatar lowers its arm into the 3D thigh or the arm level in the horizontal position is wrong. In future, the individual anatomical proportions should be computed from the data of the motor task using the existing knowledge of the initial position and the integrated distance of lifting the hand from thigh to horizontal position. It has to be evaluated whether the data should be scaled to the avatar using a mathematical model of the motor task, or the dimensions of the avatar should be adapted to the patient’s proportions.

Finally, it should be noted that the animation of the avatar has more degrees of freedom than can be estimated by the sensor units used. Hence it is an underdetermined problem. In a real life setting, it is assumed that the PD patients wear as few sensor units as possible to increase convenience. Presumably, patients will wear four sensors on the limbs and one at the hip. Hence, animation of a patient’s movement with MARG sensor data is only possible when the patient is performing predefined movements, where assumptions can be made about the posture and the movement. For the given case of the pronation-supination task, the movement of the upper arm has to be estimated by the motion model. For the presented application, namely the animation of UPDRS exercises, the avatar is suitable and animation is possible.

## 5. Conclusions

In summary, both the developed classification model as well as the avatar have shown good evaluation results for the pronation-supination motor task. The mean agreement between: (a) the ratings based on video and (b) the automatically classified UPDRS is 0.48 and (c) the 3D avatar is 0.47. The ratings from (b) and (c) are comparable with ratings based on video recordings. Thus, the classifier can be used for a quantitative overview of the monitoring period and the avatar is similarly suitable for assessing the UPDRS as video recordings. In the future, we plan to optimize the avatar by adding shadows to the animation and to omit the upper arm sensor, replacing it with a model to estimate the upper arm movement. The suitability of the avatar will be assessed by implementing more UPDRS exercises, including exercises that involve lower extremity movement, as well as whole body movements like sit-to-stand task. These additions and improvements will then be evaluated in a follow-up study, involving a larger set of patients and control subjects as well as neurologists for assessment. The data sets obtained during this study will, in turn, be used for evaluation and the further development of the classifier.

## Figures and Tables

**Table 1:** Study population.

Characteristic	PD Patients	Controls
n	13	13
Age (years, mean ± SD)	66.6 ± 9.72	66.15 ± 10.8
Sex (male:female)	9:4	7:6
Disease Duration (years, mean ± SD)	8.2 ± 4.62	-
PDQ-8	41.7 ± 12.79	-
UPDRS Task 3.6 (mean ± SD)	2.02 ± 1.16	0.51 ± 0.83

**Table 2:** Definition of task 3.6 “Pronation-Supination Movements of Hands” [19].

Score	Rhythm (Interruptions)	Speed	Amplitude Decrement
0	No interruptions	Normal speed	No decrement
1	1 to 2	Slightly slowing	Near end of sequence
2	3 to 5	Mild slowing	Midway in sequence
3	>5	Moderate slowing	Starting after first sequence
4	Cannot or can only barely perform the task		

**Table 3:** Description of features finally used for the classification.

Feature	Description	Pearson ^1^	Correspondence
mean_angRate	Mean of maximum angular rates in x-axis of all oscillation segments	−0.67	Average overall speed ^2^
median_rotAngle	Median value of maximum rotation angles from left turn to right turn of all oscillation segments	−0.66	Average amplitude
upQuart_rotAngle	Mean value of the upper quartiles (75%) of the rotation angle within all oscillation segments	−0.63	Amplitude
ratioQ13_angRate	Ratio of the mean angular rate in the first third of oscillation segments to the last third of segments	0.51	Decrement of speed during test
ratioQ13_rotAngle	Ratio of the mean of the rotation angles for a full turn of the hand in the first third of oscillation segments to the last third of segments	0.20	Decrement of amplitude during test ^2^
std_rsquare_1n	Standard deviation of *R*² values for single-term sine fit in the oscillation segments	0.70	Rhythm ^2^
mean_rsquare_3n	Mean of *R*² values for three-term sine fit in the oscillation segments	−0.60	Smoothness and regularity of pronation-supination
std_rsquare_3n	Standard deviation of *R*² values for three-term sine fit in the oscillation segments	0.59	Smoothness and regularity of pronation-supination

^1^ Pearson correlation coefficient between the feature and the UPDRS score; ^2^ These criteria are defined exactly in the MDS-UPDRS item 3.6 (see Table 2).

**Table 4:** Training and evaluation data.

Characteristic	Training Data	Evaluation Data
Number of Data Sets n	86	15
Subjects (PD patients:controls)	13:13	10:5
Age (years, mean ± SD)	66.12 ± 10.08	65.07 ± 9.67
Sex (male:female)	16:10	9:6
UPDRS rating (mean ± SD)	1.15 ± 1.27 [0 to 4]	1.67 ± 1.29 [0 to 4]

## Abbreviations

The following abbreviations are used in this manuscript (listed by appearance in text):

PD	Parkinson’s disease
UPDRS	Unified Parkinson Disease Rating Scale
MDS	Movement Disorder Society
MARG	magnetic, angular rate (gyroscope) and gravity (accelerometer)
PDQ	Parkinson’s Disease Questionnaire
SVM	Support Vector Machine
SD	standard deviation
*R*²	coefficient of determination
ANOVA	Analysis of Variance
BMI	body mass index

## Appendix A

On Zenodo you find the following supplementary data:

- Description and mathematical details about all 51 features
- anonymised data about the subjects (age, gender, PD patient/control group)
- 51 data features for each record
- results of the different UPDRS ratings from all neurologists and the classificator
- raw data of the pronation-supination phase in single data files (csv)

## References

[1] Braak H., Del Tredici K. (2009). Neuroanatomy and Pathology of Sporadic Parkinson’s Disease (Advances in Anatomy, Embryology and Cell Biology; 201).

[2] Hughes A.J., Daniel S.E., Kilford L., Lees A.J. (1992). Accuracy of clinical diagnosis of idiopathic Parkinson’s disease: A clinico-pathological study of 100 cases. J. Neurol. Neurosurg. Psychiatry.

[3] Movement Disorder Society Task Force on Rating Scales for Parkinson’s Disease (2003). The Unified Parkinson’s Disease Rating Scale (UPDRS): Status and recommendations. Mov. Disord. Off. J. Mov. Disord. Soc..

[4] Post B., Merkus M.P., de Bie R.M.A., de Haan R.J., Speelman J.D. (2005). Unified Parkinson’s disease rating scale motor examination: Are ratings of nurses, residents in neurology, and movement disorders specialists interchangeable?. Mov. Disord. Off. J. Mov. Disord. Soc..

[5] Patel S., Lorincz K., Hughes R., Huggins N., Growdon J., Standaert D., Akay M., Dy J., Welsh M., Bonato P. (2009). Monitoring motor fluctuations in patients with Parkinson’s disease using wearable sensors. IEEE Trans. Inf. Technol. Biomed..

[6] Klucken J., Barth J., Kugler P., Schlachetzki J., Henze T., Marxreiter F., Kohl Z., Steidl R., Hornegger J., Eskofier B. (2013). Unbiased and Mobile Gait Analysis Detects Motor Impairment in Parkinson’s Disease. PLoS ONE.

[7] Giuberti M., Ferrari G., Contin L., Cimolin V., Azzaro C., Albani G., Mauro A. Linking UPDRS Scores and Kinematic Variables in the Leg Agility Task of Parkinsonians. Proceedings of the 2014 11th International Conference on Wearable and Implantable Body Sensor Networks (BSN).

[8] Giuberti M., Ferrari G., Contin L., Cimolin V., Cau N., Galli M., Azzaro C., Albani G., Mauro A. On the Characterization of Leg Agility in Patients with Parkinson’s Disease. Proceedings of the 2013 IEEE International Conference on Body Sensor Networks (BSN).

[9] Giuberti M., Ferrari G., Contin L., Cimolin V., Azzaro C., Albani G., Mauro A. (2015). Assigning UPDRS Scores in the Leg Agility Task of Parkinsonians: Can It Be Done Through BSN-Based Kinematic Variables?. IEEE Internet Things J..

[10] Printy B.P., Renken L.M., Herrmann J.P., Lee I., Johnson B., Knight E., Varga G., Whitmer D. Smartphone application for classification of motor impairment severity in Parkinson’s disease. Proceedings of the 2014 36th Annual International Conference of the IEEE Engineering in Medicine and Biology Society.

[11] Heldman D.A., Filipkowski D.E., Riley D.E., Whitney C.M., Walter B.L., Gunzler S.A., Giuffrida J.P., Mera T.O. Automated motion sensor quantification of gait and lower extremity bradykinesia. Proceedings of the 2012 Annual International Conference of the IEEE Engineering in Medicine and Biology Society.

[12] Mera T.O., Heldman D.A., Espay A.J., Payne M., Giuffrida J.P. (2012). Feasibility of home-based automated Parkinson’s disease motor assessment. J. Neurosci. Methods.

[13] Cancela J., Pansera M., Arredondo M.T., Estrada J.J., Pastorino M., Pastor-Sanz L., Villalar J.L. A comprehensive motor symptom monitoring and management system: The bradykinesia case. Proceedings of the 2010 Annual International Conference of the IEEE Engineering in Medicine and Biology.

[14] Cancela J., Pastorino M., Arredondo M.T., Konstantina N.S., Villagra F., Pastor M.A. (2014). Feasibility Study of a Wearable System Based on a Wireless Body Area Network for Gait Assessment in Parkinson’s Disease Patients. Sensors.

[15] Keijsers N.L., Horstink W.W., Gielen S.C. (2003). Automatic assessment of levodopa-induced dyskinesias in daily life by neural networks. Mov. Disord. Off. J. Mov. Disord. Soc..

[16] Stamatakis J., Ambroise J., Crémers J., Sharei H., Delvaux V., Macq B., Garraux G. (2013). Finger tapping clinimetric score prediction in Parkinson’s disease using low-cost accelerometers. Comput. Intell. Neurosci..

[17] Khan T., Nyholm D., Westin J., Dougherty M. (2014). A computer vision framework for finger-tapping evaluation in Parkinson’s disease. Artif. Intell. Med..

[18] Piro N.E., Baumann L., Tengler M., Piro L., Blechschmidt-Trapp R. (2014). Telemonitoring of Patients with Parkinson’s Disease Using Inertia Sensors. Appl. Clin. Inform..

[19] Christopher G.G. (2008). MDS-UPDRS. http://www.movementdisorders.org/MDS-Files1/PDFs/Rating-Scales/MDS-UPDRSfinal_Update.pdf.

[20] Piro N.E., Piro L.K., Kassubek J., Blechschmidt-Trapp R.A. (2016). Parkinson Research: MARG Sensor Data of the Pronation-Supination Task. Zenodo.

[21] Jenkinson C., Fitzpatrick R., Peto V., Greenhall R., Hyman N. (1997). The PDQ-8: Development and validation of a short-form Parkinson’s disease questionnaire. Psychol. Health.

[22] Peto V., Jenkinson C., Fitzpatrick R. (1998). PDQ-39: A review of the development, validation and application of a Parkinson’s disease quality of life questionnaire and its associated measures. J. Neurol..

[23] Madgwick S.O.H., Harrison A.J.L., Vaidyanathan A. Estimation of IMU and MARG orientation using a gradient descent algorithm. Proceedings of the 2011 IEEE International Conference on Rehabilitation Robotics.

[24] Witten I.H. (2005). Data Mining: Practical Machine Learning Tools and Techniques.

[25] Quinlan J.R. (1993). C4.5. Programs for Machine Learning.

[26] Zechner M. libGDX 1.5.4, 2015. libgdx.badlogicgames.com.

[27] MakeHuman Team MakeHuman 1.0.2. http://www.makehuman.org/.

